# Supplementary material for: Further evidence for the capacity of mirror self-recognition in cleaner fish and the significance of ecologically relevant marks
Source: PLoS Biol. 2022 Feb 17;20(2):e3001529. doi: 10.1371/journal.pbio.3001529 (PMC8853551; doi:10.1371/journal.pbio.3001529)
Supplement: Attachment — Submitted filename: Reply to PLOS Biology. Final.docx [file pbio.3001529.s002.docx]

4 September 2021

Dear, Editor of PLoS Biology

Here we made point-by-point response letter to reviewers. Our answers are shown in red, and this letter show almost all Line numbers of sentese we improved.

We give text file showing changing points. The line numbers of sentence in the current ms are shown in point-by-point response letter.

Best wishes

Masanori Kohda

We will show point-by-point response as followings.
*****************************************************
REVIEWERS' COMMENTS:

Reviewer #1:
[identifies himself as Prof. Culum Brown]

This is a very comprehensive study of the MSR test in cleaner fish and addresses many of the potential criticisms put forward by critics of a previous paper. Importantly, the sample size is now substantial. It would be fair to say that the cleaner fish is now one of the most extensively studied animals in the context of this test outside of primates.  For me, many of the previous criticism were simply not valid, but the authors of the current paper have addressed them none-the-less.  Perhaps the most important outcome of this paper is the notion that the mark must be salient and or ecologically relevant to the species. Fishes make an excellent model system with which to test this hypothesis further. It is quite clear that familiarity with a mirror is also very important so the subjects learn contingencies between their own movements and their reflections.

On the whole, the paper is well presented and easy to follow. In some instances the language is a little awkward. I've tried to correct some of these cases, but it may be beneficial for the type setters to fix some of the grammar.  I felt the language was a little conservative in places, I believe the authors have good reason to be more assertive about these findings.

Reply: Thank you very much for this positive feedback. There will be further follow-up studies testing new aspects of MSR. For the moment, we prefer to keep our interpretation of the results rather neutral. As referee 3 points out, none of the extra experiments provides conclusive positive evidence; they are mainly designed to provide negative evidence if subjects ‘misbehave’.

Specific comments:
L41-42: Or it is possible that fish are self-aware. Ie the test is not broken, it really does what we think it does and fish passed.

Reply: We agree of course. The following sentence with underline is the L43-44. We still take moderate expression similar to our previous paper, because the level of the present studies will be similar to that of the previous paper, instead of much sample size and many positive results from additional tests (Kohda et al. 2019).

Therefore, we reiterate the conclusion of the previous study that either self-awareness in animals or the validity of the mirror test needs to be revised.

L107: Surely the fact there was no response to the invisible elastomer counters this argument.

Reply: Yes, of course, we agree with your suggestion. To show no physical sensation by marking with injection, we did this invisible sham mark-test. However, the critics by Gallup & Anderson and de Waal is when the visual stimulus of color marking and implant mark are perceived at the same time, fish will feel physical stimulus, but only with injection stimulus fish will not feel pain. From their comments, no response to the invisible elastomer will not be a control.

the possibility that the elastomer marks also produced some physical sensation, akin to the head implant in rhesus macaques.

L167-168: Clearly this isn't the case with elastomer. The colour is irrelevant to the final texture.

Reply: Yes, we think so. This is also related to the two critics: when fish do not see the mark-color they will not feel pain. Unfortunately, most readers of this paper will not understand elastomer marking.

argued that the transparent sham-mark may be different enough so that cleaners do not feel its presence.

L185: Ecologically relevant in as much as the attention of the subject is drawn to the mark. But this does not invalidate the test. It does, however, provide some important information about why some animals / individuals fail.

Reply: Yes, you are right. The ecological relevance does not reduce the value of the test, and this implies the reason why some animals/ individuals fail in many previous studies. Our experiment using ecologically non-relevant mark of blue and green suggest that ecologically irrevant mark will induce the opposite results and conclusions, and this will be the cases of many previous studies.

Only an ecologically relevant mark suggesting the presence of an ectoparasite induced throat scraping.

L196: This reminds me of the behaviour Sneddon and others observed when injecting bee venom and or acid into the lips of fish (trout from memory) to study pain responses.

Reply: We also think it is similar to our test.

The result shows that a painful or itching mark does not require a mirror to elicit self-scratching.

L231: I don't understand why the mark speeds up learning. I understand that prior exposure the mirror enables the fish to realise that the mirror image is not a threat / conspecific and perhaps later that its actually its own reflection.

Reply: We agree that the result is puzzling. It is clear that there was a significant difference in frequencies of mark scraping between the normal marking and this control marking, the latter shows much lower frequencies, supporting our prediction. However, we had assumed fish achieve MSR after several days, and the reason of the much earlier timing of scraping mark is not sure. One possible reason we think is the mirror self-recognition can be originally much earlier than the proposal by Kohda et al. 2019, and this will also be so in chimpanzee, dolphins and elephant probably. This possibility is beyond the scope here, and we cannot make such discussion in the present MS, and will write in a future paper on this matter with more data from additional experiments.

Thus, now we will change the sentence as follows.

It would be difficult to assume that the speed of learning the mirror contingencies seemed be enhanced by the subjects seeing the mark in the mirror. To consider the cause fairly, we need quantitative data of mirror-related behavior of non-marked naïve fish in the initial one or two hours after mirror setting, but not have it. More importantly, however, New Lines: 241-245.

whether the marked cleaners learned the mirror contingencies atypically fast or whether . As it stands, Gallup (1970) tested only two mirror-naïve individuals during 30 min of exposure.

L242: whether feedback between a visual and a physical stimulus may cause throat scraping

Reply: We deleted ‘a’ prior to feedback.

as an additional way to test whether a feedback between a visual and a physical

L268: No it may be they are just familiar with the reflected image. Suggest you ref one of the many studies of familiarity effects in fishes.

Reply: Dear neighbour effect will be just the one, and we refer such study of our own. (e.g., Kohda et al. 2015)

Note, however, that the lack of aggression by itself does not show that cleaners recognize the mirror image as self. Alternatively, the subjects could have habituated to what they perceive as one specific other individual, and hence show no aggression no matter where they encounter it (e.g., Kohda et al. 2015).

L321: Worth noting that there are several species of cleaner fish, and other species are known to "peck" at dots with high contrast, presumably because they mimic food. So there is ample opportunity to test the "ecological significance" in a range of fish species.

Reply: Yes, thanks. As you say, the next study should include subjects of other cleaning fish species, and show the significance of ecologically relevant mark.

L379: It is implied in the text that the fish are wild captured, but here you state they are from fish shops. It needs to be clearly stated that fish shops collect them from the wild.

Reply: Yes! Fish shop imports cleaner fish directly from South-east Asian countries, mainly Philippine and Indonesia. The stuff of ‘ornament fish export company’ capture coral reefs and export them immediately and periodically. So, we rewrite the sentense as follow:

We used a total of new 35 wild captured cleaner fish obtained via commercial ornament fish shops. [from to via] New Line 404

L430: I have to say that I personally have marked 1000s of fish using VIE and I have never once seen them scrape in an attempt to remove it. I have used every colour on offer. So the notion that the colour of the mark might change how the injection feels to the fish seems remote in the extreme.

Reply: We have the same experiences. These would be strong enough to show the no effect of the color mark by injection. But most readers study animals other than fish…..

L443: "did not touch the work" is an odd phrase.

Reply: Thanks. We change it into `did not conduct the experiments’ (New Line 490)

L447: "as reported…"

Reply: we changed so. (New Line 509)

L492: "apparently did not…"

Reply: We changed. (MS L564)

L508 " and we marked… following the standard marking"

Reply: Thanks! (New L580)

To Reviewer #1, thanks for appreciating the MS and many helpful and constructive comments that will help us with future research.

Reviewer #2:

This is a possibly highly interesting and possibly quite inspiring study. However, while the information on the manipulations done on the experimental subjects as well as the information on the experimental setting is sufficient, there is a serious lack of information on a most crucial part of this study: How, exactly, were the behaviours of the fish observed and rated?

In experiment 1, there were three 'independent' observers. What did they do? Did each of them independently analyse a subset of trials? In such experiments it would be mandatory by today's standards to have a complete and independent rating of the footage by each of at least two observers and subsequent calculation and reporting of some measure for interobserver reliability. Ideally, the raters would not know the aim of the study, for example, they would not be informed about which of the colour conditions was a critical test with an expected increase in scratching, and which of the colour conditions were just controls.
Of course, based on the manuscript it cannot be excluded that the authors have been following such experimental standards. If so, it would be absolutely necessary to report this in detail.

Reply: As Rev. 2 says, for blind test, the worker (rater) should not know the aim of the study and any information related to each experiment. But in *Exp. 2,* the workers need to directly observe blue, green and brown marks on subject fish, and in this case to make a complete blind test is difficult. However, the throat scraping behaviors are salient, and the occurrence of throat scraping behaviors is easily identified from the other behaviors and scraping of other body parts, and the identifying this behavior from video is not difficult. We now show the inter-observer reliability scores on throat watching duration more difficult in quantitative observations than numbers of mark scraping.

Fujita and Sogawa observed 8 fish that had only brown mark in *Exp. 1*, and Kubo and Sogawa observed 6 fish with blue, green and brown marks in *Exp. 2*. Each team conducted, setting of experiment tank, video recording, VIE-marking, Video analyses. After these recording, Kohda checked the videos of all events of throat scraping, and confirmed them as throat scraping.

Kubo also analyzed throat watching duration after VIE injections of three color-mark. This observation of watching reflection within 5 cm from mirror were more difficult than count the numbers of scraping throat.

These detail of the observations and conditions are described in Material and Methods.New Lines: 442-445, 491-506, 535-541.

The fighting time duration and frequencies of contingency behaviour during the first one week before mark-test of 8 fish in *Exp.1* are newly analyzed by Kubo, for revision of the MS. (New Line 444-445)

There is another serious shortage in the behavioural data: Findings on two critical indicative behaviours are reported without any frame of reference provided by the overall activity or specific other activities. Overall, the reported behavioural frequencies are quite low. Therefore, it has to become clear from the data that the difference between critical test conditions and various control conditions cannot be caused by a general increase in behavioural activity. Without any information on other behavioural activities an evaluation of this essential point is not possible. Preferably, the reported frequencies of mark-directed behaviours would be expressed as a percentage of overall activity. At least, this measure or a similar measure should be added.
As all experimental sessions have been videotaped, it should be no problem for the authors to obtain data for additional controls (e.g. interobserver reliability) and for behavioural contextualisation within a reasonable amount of time.

Reply: We agree with Rev. 2 that the frequency of mark scraping is very low. As the videos show, the acts are short. Thus, occurrence data seem to be appropriate to present the events. One likely reason why event frequencies were low is that the fish do not see the mark unless they swim up when in front of the mirror. The experiment with the marks of three different colours shows that the same physical treatment causes significant changes in swimming up in front of the mirror and in throat scraping as a function of the color of the mark. If marks are blue or green, throat scraping is entirely absent (rather than reduced). Otherwise, the small size of the aquaria caused subjects to be permanently exposed to the mirror while swimming around in the tank (especially as the fish have large fields of vision). It is not like in other studies that subjects could avoid seeing themselves.

Regarding the reliability of interobserver, we made additional control test, the blind test. To test reliability of behavioural observation, a complete blind test was conducted. A set of 25% (2 of 8 fish in *Exp. 1*) of the video with no information of date or hour and fish name was analyzed by a researcher outside our team as a blind-test, and the results were compared with the original results by Kubo. These data set was highly correlated (LLM, R^2^=0.991, F=1250, p < 0.001), and the statistical tests showed strong reliability of the original data (by Kubo with date information). These are written in Materials and Methods in text. New Line: 502-508 in text.

Fig. 3 shows the time in posturing throat mark of control (no), blue, green and brown mark, during 600 seconds (10min) observations in a), and mark scraping frequency in the four tests. Each of 6 fish experienced the 4 tests in random order, and 4 conditions experiment tanks and conditions of each test were the same. The difference was just the time posture reflecting the throat (a), and frequency of throat scraping (b). As Rev. 2 recommends, we will describe other behaviors of fish against mirror in Fig. 4 and Table 1 in Method briefly.

‘There is another serious shortage in the behavioural data: Findings on two critical indicative behaviours are reported without any frame of reference provided by the overall activity or specific other activities.’

We refered Kohda et al. 2019 when it is necessary and explain the contingency-checking behavior (atypical behaviors), self-directed behaviour (i.e., frequent watching its refelection and mark-scraping behaviour).

Reviewer 3 also says that fish behavior data before mark-test should be shown. For this purpose, Kubo newly checked the video-recordings of the first one week after presentation of mirror in. *Exp. 1*, and recorded fighting duration and frequencies of contingency behaviors and the duration of watching its face and/or body within 5 cm from the mirror surface, that is the only self-directed behavior found in this fish prior to mark-test, were observed. The daily changes in data are shown in supplementary Fig. 4 in revised MS. Also, the definition of these contingency-testing behaviors is given in Table 1 and methods.

To test reliability of behavioural observation, a complete blind test was conducted as mentioned above. The statistical tests between original observation and complete blind observations showed strong reliability of the original data (by Kubo with date information). These are written in Materials and Methods in text. New Line, 501-506.

Reviewer #3:
[identifies herself as Diana Reiss]

Review of PBIOLOGY-D-21-00901R1- Further evidence of mirror self-recognition of the cleaner fish, and the significance of ecologically relevant marks

The authors state that they conducted the current study to address some of the criticisms and what were suggested as "shortcomings of the Kohda et al 2019 study and they have conducted 6 experiments to do so. In their abstract they summarize their results as follows: "1) 14/14 new individuals scraped their throat when a brown mark had been provisioned, but only in the presence of a mirror, 2) Blue and green colour marks did not elicit scraping, 3) Intentionally injecting the mark deeper beneath the skin reliably elicited spontaneous scraping also in the absence of a mirror, 4) Mirror-naïve individuals injected with a brown mark scraped their throat with lower probability and/or lower frequency compared to mirror-experienced individuals, 5) In contrast to the mirror images, seeing another fish with the same marking did not induce throat scraping, 6) Moving the mirror to another location did not elicit renewed aggression in mirror-experienced individuals." 

I found the results reported on the responses of the cleaner wrasse in this paper and the previous paper by Kohda et al. 2019 very interesting. However, I remain unconvinced by the findings in this report that the wrasse of showing behavior that is equivalent to the results reported in apes, dolphins and elephants. However, the present study has been well-designed to address some of the criticisms and shortcomings of the previous study and indeed the new results are also very interesting and important to help clarify the factors that may contribute to our understanding of why the fish are scraping the marked area of their bodies in the mirror condition more frequently than in the absence of the mirror. In my comments below I will explain the reasons why I remain unconvinced from the results reported that the cleaner wrasse perceive that the image in the mirror is themselves.

Reply: We thank the referee for her insightful comments. We agree in the sense that the new tests were more designed to disprove MSR if the fish had behaved in the wrong way rather than providing positive evidence (where the mark test is simply the best one). Nevertheless, the fact none of the new results contradicts the MSR hypothesis should increase our confidence in the interpretation by Kohda et al. 2019.

For the reasons outlined above, we think that discussing all alternative interpretations for each single test goes beyond the scope of the ms. Instead, we outline the logic of our approach in the new discussion, stating explicitly that the experiments were designed to challenge the MSR interpretation, where repeated failure to do so is indirect positive evidence. Nevertheless, we also reply to each comment below individually.

Experiment 1: Replication of mark-test. During the first five days, fish initially exhibited aggression, and contingency-testing behaviors but these decreased and were hardly observed by seven days as in the previous paper (Kohda et al. 2019). 

Lines 406-409: "This latter phase is indicative of subjects having recognized the mirror- reflection as themselves and hence being ready to pass the mark test (Kohda et al. 2019). Thus, in the present study we started to test subjects after one week of mirror presentation, if they exhibited these reactions (Kohda et al. 2019)." One of the main issues I have with this paper is the lack of qualitive and quantitative data and descriptions of the specific behaviors the wrasse exhibit during mirror exposure prior to the mark test. Notably, in past MSR studies with other species (apes, dolphins and elephants) prior to the mark-test, these animals show a variety of self-directed behaviors (not just one behavior). If we consider our use of a mirror, we learn to interpret the information in the mirror based on learning the contingencies of mirror use and as previously described in the MSR literature, mirror naïve apes, dolphins and elephants, show a progression of stages of behavior that are observable reflections of the perceptions (stage 1 social behavior/ mirror exploratory behavior; stage 2: contingency testing (unusual and/or repetitive behaviors); stage 3; self-directed behaviors (and this is a critical stage as the animals seek out the mirror and use it as a tool in viewing parts of their body not-viewable in the absence of a mirror. It is this behavior—that is a first evidence and critical evidence of MSR - not just the touching or response to the mark.  The focus on the mark test makes it very difficult to interpret the results as presented.

Reply: As the cleaners’ behavior during the three stages had been described previously (Kohda et al. 2019), we had thought that referring to this paper would have been sufficient. Nevertheless, we will add information on the cleaners’ behavior during the first 7 days in Materials and Methods (Fig. 4), with the description of contingency-checking behaviors (Table 1) and self-directed behaviorsp by using video data of Exp. 1. We describe the patterns of aggressive behaviour, contingency-testing behaviour and self-directed behaviors in detail as in the previous paper (but self-directed behavior of this fish before marking is simple, that is, watching their mirror-reflection near the mirror). As the referee recommended, we analyzed these behaviors in function of daily change, and make a list and individual difference of the contingency behaviour, which are shown in Fig. 4 and Table 1 in revised MS. New Line, 440-453.

Lines 151 -157: There is an error in the following statement that needs to be corrected. The authors state that the "Cleaner wrasse also currently show the highest rate of passing (94% = 17/18; and one failing fish in Kohda et al. 2019) is incorrect and then list all the other studies that have a lower % except the two studies that report a higher %. Specifically, the authors fail to cite the dolphin studies (Reiss & Marino, 2001 in which 2/2 dolphins passed multiple mark tests and Morrison & Reiss, 2018 in which 2/2 dolphins passed the mark test (4/4 =100%).

Reply: Yes, it was of our mistake. Now, (4/4 = 100%) is described in these lines. New Line, 172-173.

In contrast, only a small proportion of individuals pass the test in the animal species of MSR-capacity reported hitherto, e.g. ca. 40% in chimpanzee (n = 97), 50% in orangutans (n = 6), 30% in gorillas (n = 15), 30% in Asian elephant (n = 3) and 40 % in magpie (n = 5) (Povinelli et al. 1993; Swarts and Evans 1991; Swartz et al. 1999; Prior et al. 2008; Suddendorf and Collier-Baker 2009), except 100% in dolphins (n = 4) (Reiss and Marino 2001; Morrison & Reiss 2018).

Experiment 2: Green and blue marks do not elicit scraping: Only the brown ecologically relevant mark elicited throat scraping and the authors conclude: "This experiment demonstrates that visual information - with or without a potential physical stimulus from the injection - is not enough to elicit throat scraping. Instead, it appears that the visual stimulus needs to be salient and of negative valence. Only an ecologically relevant mark suggesting the presence of an ectoparasite induced throat scraping".  Lines 465-469- If ecological relevance of the mark is important, we expected cleaners to scrape their throat more frequently and to spend more time posing to the mirror (assuming a vertical position exposing the mark to the mirror) when the mark was brown compared to blue and green marks (Kohda et al. 2019).

The finding that the fish positioned more frequently to the brown mark is evidence of the ecological relevance and saliency, as predicted by the authors, but as suggested in previous criticisms, the saliency of the brown mark as opposed to the other marks could result in the positioning based on learning the contingencies of mirror use -moving the body in specific ways results in seeing the mark.  Therefore, it is critical to have a behavioral record and description of the types of behaviors, contingency testing and self-directed, that the fish exhibited during mirror exposure. Again, focusing on the mark-directed behavior only in the absence of a detailed account of the behavior during mirror exposure makes it difficult to interpret the results and weakens the claim for MSR in the cleaner wrasse.

This fish showed aggression to reflection, contingency-testing behaviour during the first week, and showed self-directed behavior (wachting themselves in mirror near the mirror) was observed after 5 days (Fig. 4, Table 1). The experiments with color-marking started after 10 days, when the fish swam normally like before the experiment, and did not exhibit specific actions.

The results of this mark of different colors showed fish check the brown mark more frequently than blue or green mark (Fig. 3). Fish started the throat checking after the mirror exposure, with higher frequency against brown mark. This strongly suggest there will be few chances for specific associated-learning only for this color mark. Alternatively, the plausible explanation will be that fish find specificity in the brown color, that is, the ectoparasite they experimenced in the wild habitats, whereas color not resembling parasites are assumed not harmful.

As reviewer 2 says, we also question the low frequency of the mark scraping. We can think of various potential explanations, one being that the mark is not visible most of the time. We observed that after scraping the throat mark, fish immediately (within 2 sec) attempt to check the throat of the scraped mark, as if to check whether or not the mark is removed. One interpretation of the low frequency of the scraping is that after scraping mark they will see the mark remaining still but with no physical sensation. Then fish will give up to remove it or regard the mark is not parasite, resulting in a few times of mark scraping probably. Another interpretation will be that fish regard the brawn mark as parasite-like mark but not real parasites from the beginning. So, they did not scrape it frequently. But anyway, it will be said assertively when fish see the brown mark, they give more attention to the parasite-like mark than other color marks. These will be difficult to discuss because of lack of data related to this problem.

Of course, we understand the importance of description of contingency-testing behaviour and self-directed behaviour before mark-test. In the previous paper (Kohda et al. 2019), we described these behaviors in detail including throat scraping behaviour (self (mark)-directed behaviour). The self-directed behaviour of this fish that have no limb and fingers (like dolphins) is simple: only watching their reflection close to the mirror prior to mark-test (Kohda et al. 2019). This watching appears constantly after 5th day of mirror presentation (Fig. 1A in Kohda et al. 2019). However, here we will describe these behaviours based on video-recordings in the present study, and these data which will assist to understanding the social behavior (Stage 1), contingency behavior (Stage 3) and self-directed behaviour (Stage 3) of this fish (Table 1 and Fig. 4). New Line, 427-453.

This is an interesting result and as the authors suggest it supports the view that the fish are attending to only the ecologically relevant brown marks. One question I have is whether the blue and green marks are visible on the bodies of the wrasse in the mirror refection?  Would the wrasse attend to these marks at all in their natural environment? The fish only scraped the marked area in the presence of the mirror and as the authors report, either the visual mark in the mirror or that the combined visual and physical sensation trigger the scraping response.

Reply: Yes, the cleaner fish have very good eyes, and they can distinguish varieties of colorations including red, orange, yellow, green, blue, white and black, which have been experimentally tested (Hotta et al. 2020) as we described in the text. So, the sentence below was put into text New Line 512-513.

Cleaner fish identify a variety of color including blue and green (Hotta et al. 2020).

In some experiments, fish scraped the mark on the throat, but such throat scrapings have not been observed in coral reefs (Kohda and Bshary personal observation). Additionally, fish never show an attempt to remove the VIE-mark in any fish species and individuals in many practices (pers.obs., Kohda and Bshary, see also the comment by referee Culum Brown). Throat scraping only after seeing mark resembling parasite in mirror must be mark scraping behaviour without physical stimulus, we think.

If visual stimulus of color mark will elicit the physical stimulus of mark (Gallup and Anderson 2020, de Waal 2019), green and blue as well as parasite-like color mark brown must elicit the painfulness, and fish will scrape the mark because of elicited physical stumulus. However, as we showed, subjects did not scrape the green and blue marks. If a color dot on the injection point makes cleaners remember the presence of a physical stimulus, green or blue marks should also be scraped.

Hotta et al. 2020. (Hotta T, Ueno K, Hataji Y, Kuroshima H, Fujita K, Kohda M (2020) Transitive inference in cleaner wrasses (*Labroides dimidiatus*). Plos One, 15: e0237817.)

Experiment 4:  Mirror-naïve individuals injected with a brown mark scraped their throat with lower probability and/or lower frequency compared to mirror-experienced individuals. This seems to support the view that the sensation of the brown mark elicits some scraping but to a lesser degree in the absence of the mirror and that the visual and physical sensation increase scraping. This result is similar to the results reported by Rajala et al. 2010 and further supports the view that the combination of the two sensory inputs, visual and physical sensation may focus the animal's attention and lead to understanding the mark is on self. However, it is important to state that this is not required for the apes, dolphins, or elephants in the past studies as the animals as neither the mark nor sham elicited a mark-directed response prior to or in the absence of the mirror. These differences are important in the comparison of results and how MSR is described across species (see Reiss and Morrison (2017). 

Reply:　In *Exp. 3*, deeper injection apparently provided physical stumulus, and fish scraped the mark in the absent of mirror. So, this result is not against capacity of MSR of this fish. But the normal marking by subcutaneous injection does not give physical stumulus, as we showed in *Exp. 2.* If visual plus physical stimulation was important, blue and green marks should have elicited scraping as wel, but never (*Exp. 2*). In contrast, the deeper injection should have led to scraping without or with mirror. Fish do not need a mirror to scrape their throat if it itches.

The combination of physical and visual stumulus will not be the case of cleaner fish, and are not similar to Rajala et al. 2010, and the other monkey experiments using a monkey chair and laser beam. As we showed, the normal injection of VIE will not give any physical stumulus to fish, and the mark-test will be very similar to the cases of ape, elephants and dolphins: cleaner fish did not show mark-directed behaviour to sham-mark in the presence of mirror, and fish show mark-directed behavior to color mark only in the presence of a mirror, never in the absence of mirror (*Exps. 1, 2* and Kohda et al. 2019).

We point out in the discussion that data such as ours (naïve fish with mark in an initial hour) do not exist for other species; the control mark-test of providing mark to mirror naïve fish was only conducted in two chimpanzees for 30 minutes. Only if studies on other species show that markings do not speed up learning our data can be interpreted as the referee suggests. Currently, the other results of the study remain: mirror-naïve cleaners are less likely to scrape their throat and if they do then with lower frequencies.

Their mark scraping by marked naïve fish occurred significantly earlier than our prediction. We have a new hypothesis that MSR will occur much earlier not only in the cleaner fish but also in chimpanzee (Prof. Gallup and I regard the MSR will be achieved a few days or several days later after mirror presentation), probably also in elephant and dolphins (standard MSR-study does not observe animal resposes to mirror within the first one hour intensively). To test the hypothesis, we now start the related experiment (intensively observing the reaction against mirror of non-marked naïve fish within the initial two hours, which will provide data that will contribute to the understanding MSR in animals. The hypotheses and supportative data will be published in a future paper, but to expain detail is difficult now and beyond the scope of the current study. (New lines, 241-245, lines, 320-330)

As the reviewer recommends, for understanding MSR of this fish, we described the contingency-testing behaviour and self-directed behaviour of fish in the new text and in Fig. 4 and Table 1. (New lines, 427-453)

Experiment 5. Again, it is hard to interpret and conclude much from the results presented. As I previously mentioned a more complete description of the behavioral responses of the wrasse toward the other fish is needed here. Did the wrasse orient to the marks on the other fish? Did they approach the wall going toward the mark on the other fish? It would be important to mark the fish when together and determine if they try to remove the mark on each other. Furthermore, as in de Waal, 2005, it was demonstrated that individuals respond differently to an individual whose behavior is synchronized with their own than to another whose behavior is not and this should be included in the discussion and the findings are consistent with those findings. However, this experiment does not provide evidence for MSR.

Reply: As we described in text, the purpose of this experiment 5) is, if seeing real cleaner fish with brown mark on the throat, not its mirror reflection with the throat mark, would somehow remind subjects of the mark on their own throat, we would expect fish scrape the throat in this experiment as well. The purpose of this experiment is against the comment that the mark on throat in mirror will remember the physical stimulus on the throat. For the purpose it will be enough to examine whether or not they do mark scraping, and we showed the clear fish never did it, suggesting fish regard the self-reflection as itself. This is the purpose of the *Exp. 5*.

We are not going to provide evidences of many aspects of MSR from this exp 5), wherein each neighbor has brown mark on its throat. Different from de Waal 2005 experiment, we conducted color mark on their throat, and each fish can see the mark on throat of the other fish but never own mark on throat. We are not trying to find fish reactions different between towards mirror and real fish, and the detailed behaviour differences are not needed to be described. The purpose of the test was to check whether seeing a mark on another real fish elicits own throat scraping, which would have been an evidence for the hypothesis that cleaners need a visual - physical stimulus combination. At the same time, we see the absence of throat scraping also as means to discard another alternative interpretation of the initial study by Kohda et al 2019, which was formulated by Prof. Gallup, i.e. that fish communicate with the neighbors, e.g., to tell to the neighbour fish by scraping its own throat such as ‘A parasite is attaching on your throat’ (Prof. Gallup in National Geography).

Rev. 3 asks ‘Did the wrasse orient to the marks on the other fish? Did they approach the wall going toward the mark on the other fish?’ We did not observe such action by subject fish toward the real neighbors which will be quite difficult to observe, and even if they try to do so, to distinguish differences between such behaviors and the others will be difficult.

Experiment 6: Fish responses to moving the mirror: The results showing that the fish did not show aggressive behavior to their mirror image when the mirror was moved to a new position (wall) in the tank was an interesting finding. The authors conclude "The results show that cleaners do not learn a spatial contingency that allows them to eventually stop aggressing their mirror image. Instead, cleaners must learn about their own individual features and selectively stop showing aggression towards these features, independently of where they see them. Note, however, that the lack of aggression by itself does not show that cleaners recognize the mirror image as self. Alternatively, the subjects could have habituated to what they perceive as one specific other individual, and hence show no aggression no matter where they encounter it. Nevertheless, the cleaners clearly outperform rhesus macaques in this particular experiment, as the latter were highly sensitive to the movement of the mirror (Suarez and Gallup 1986)."

I think there is an alternative explanation that should be included here that is more parsimonious and consistent with the results and interpretation of presented by de Waal et al (2005). As the wrasse have had ample experience seeing a fish in the mirror matching their own behavior, when they see this image at another location, it may be perceived as familiar and thus would not be responded to with aggression.  From an ecological perspective, individual recognition amongst animals is well documented. I am not familiar with the literature on conspecific recognition in wrasse and it would be very helpful to include more information about individual recognition in wrasse in this paper.

Reply: Suarez and Gallup 1986 used two rhesus monkeys that were habituated a large mirror for their ‘lifetime’ and showed a cessation of social behavior directed toward mirrors, but the monkeys showed strong aggression to their reflection in mirror set in different locations. The results indicate the possibility of just the familiarization to own reflection in a location but not MSR. The situation of mirror location will be not so different, and we can compare the results, and our conclusion will have enough reason.

This finding may also suggest that the difference in movement of the other fish in contrast to a matching movement in their own image may underlie this differential behavior - similar to the results previously reported by de Waal et al (2005) - they state "Capuchins thus seem to recognize their reflection in the mirror as special, and they may not confuse it with an actual conspecific. Possibly, they reach a level of self-other distinction intermediate between seeing their mirror image as other and recognizing it as self."  In the discussion section, the authors concur with de Waal et al 2005 that there indeed may be a continuity in self-awareness and the present study and the Kohda 2019 study may represent a case for an intermediary level of self-awareness contingent on mirror exposure.

Reply: The reactions of cleaner fish to opponents are different between mirror reflection and real fish, as in the case of capuchin monkeys - de Waal 2005. However, largely different point between capuchin monkey and cleaner fish is whether animals pass the mark-test. The cleaner fish arguably pass the mark-test, but we will not conclude the MSR of fish shows the self-awareness similar to apes, dolphins and elephant (i.e., private self-awareness) only from our results in the present paper. In this stage we cannot say MSR of this fish imply which type of self-awareness, that is, public (body) self-awareness or private self-awareness (associsated with mental state or with self-representation). This is the point Rev. 3 is unconvinced to accept, i.e. that the cleaner fish understand the mirror reflection is itself, i.e., private self-awarenss.

To discriminate two types of self-awareness in general is important, that is, to clear how animals recognize their own mirror-reflection, i.e., whether by kinesthetic -visual matching model or by self-concept model (Morin 2021). We are going to study which model will fit to the cleaner fish by arrangement of experimental design.

Discussion: Lines 293- 296 The authors state "However, the results show that the visual input must be the mirror image rather than a marked conspecific. Thus, at the minimum cleaner fish are able to learn that only the mirror image provides contingencies for self, and use this knowledge to scrape a body part with an apparent parasite attached when spotted in the mirror. The response itself, while it may appear as evidence - is not a convincing as one might think. As the authors themselves state on Lines 291-292. "We cannot exclude that there is an intermediate state of itchiness that triggers throat scraping only in combination with a visual input."

Reply: We have revised this section after discussion for several reasons. Even the markings on humans can be felt (the best studies in this respect are actually the dolphin studies we see it) and videos from the elephants and chimpanzees suggest the same. Most importantly, visual feedback did not elicit scraping when the feedback was a blue or green dot or a brown marking on another individual, i.e. all results that speak against the visual-sensory feedback hypothesis. New Line 313-317.

Another more parsimonious alternative is that the fish are demonstrating mirror-guided behavior as they have had the opportunity to learn some of the contingencies of mirror use and the ecologically important brown mark has elicited their attention to the mirror thus focusing their attention and enhanced learning. This is similar to the argument that the fish are learning the contingencies of mirror use but they do not show the level of self-recognition as evidenced in the apes, dolphins and elephant studies. The lack of convincing self-directed behavior prior to marking, weakens the authors' argument that the fish are showing equivalent cognitive behavior. I strongly suggest that this point needs be addressed and included in the paper. 

Reply: Fish show contingency-testing behaviour, which mostly occur in stage 2 (frequently 3rd and 4th days), and after a week, they hardly show such contingency-testing behaviour. We did say fish show self-directed behaviors of watching themselves near the mirror after five days by citing results in Fig. 1A in Kohda et al. 2019 and in Fig. 4 using data of *Exp. 1*. The referee knows her dolphins and is hence sure that the videos shown with the 2019 paper presented novel behaviors in supplement movies. We know cleaner fish behavior and think she simply do not see a cleaner ever turning upside down for a quick dart. We describe the contingency-testing behaviors of the subject fish in Table 1 and Fig. 4 in revised MS.

Surely, self-directed behavior is a simple one: only watching its reflection frequently near the mirror. But this will be more or less similar to dolphins before mark-provision, that also lack hands, trunk and bill. As dolphin exhibit various mark-diercted behaviour, cleaner fish exhibit many mark-directed behaviours including mark-touching behaviour, which are not observed in dolphins.

As we have already shown and in the currect paper, cleaner fish exhibit the stage 1 (social reactions to the reflection), 2 (contingency-testing behaviour) and 3 (self-directed behaviour) after mirror presentation, and then pass the mark test. These are consistent with the results of mirror tests in chimpanzee, elephant and dolphin. Self-directed behaviour before the mark test of cleaner fish is the frequent watching itself in front of the mirror (Blue line in Fig. 1A in Kohda et al. 2019; blue line in Fig. 4 in the present study). Similar to dolphin, fish have no hands, trunk, and beak. In these aquatic animals especially fish, self-directed behaviour before mark-test is simple, but after mark-test with brown color, cleaners show self-directed behaviors of scraping mark and positioning to reflect marked throat significantly long time, a self-directed behavior like dolphins. These self-directed behviour has been already said in the previous paper (Kohda et al. 2019, Line 11-14 in P7), and are now also mentioned in the present paper. The simleness of the self-directed behavior before the mark-test will not be problem.

Overall, this is an important paper as it opens up and extends our ideas and discussion regarding studies of MSR and what aspects of behavior need to be included and described to be able to conduct comparative studies across diverse species.

Reply: Thanks for your appreciation and encouragement for our study. We have already promised that we collect more data on the topic, continuing to challenge supposedly highly cognitive interpretations for the capacity of MSR of this fish.

In summary

As a cognitive scientist involved in conducting MSR studies, I am fascinated and excited about findings reporting continuities in cognitive behaviors across species. Although these are interesting results, I remain unconvinced that the wrasse understands that the image in the mirror is themselves given the scope of the results presented in this paper. There is not sufficient evidence to differentiate mirror guided behavior from self-directed behavior in the mark tests. A more detailed report and video evidence of the contingency and self-directed behaviors prior to the mark-test are needed to be able to confirm this cognitive ability in the wrasse and make the claim for MSR in the wrasse.

Reply: As already said, fish reaction of Stages 1, 2 and 3 (aggression, contingency-test behaviour and self-directed behaviors) are described in new MS (New lines: 427-453). Repeatedly speaking, the self-directed behaviors of cleaner fish before marking are very simple and few variations, but the mark-directed behaviours (of course a kind of self-directed behavior) is salient. These will be similar to the case of MSR study in dolphins.

As we say in the discussion, we are looking forward to a broader discussion of the topic, including methodological aspects. And we will present more data in the future. For example, we need how animals recognize the mirror reflection as itself, e.g., do they use kinesthetic-visual matching processes or indeed a self-concept process. Thus, we cannot say which type of self-awarenss fish have in the current papr, but we will try to show which type of self-awareness, a public self-awareness or a private self-awareness associated with self-concept, cleaner fish have in a future paper.

However, I do think that the authors have presented some very compelling evidence for mirror-guided behavior in the species and this paper is an important contribution to our understanding of comparative cognition and specifically the capacity for MSR. I hope my comments are helpful in strengthening this very interesting contribution.

Reply: Thanks for your appreciation of this MS. Your advice improves our MS. The style of the original ms is of replication and additional tests for the MSR of cleaner fish reported in Kohda et al. 2019, but now the revised MS contains important information.
