# Supplementary material for: Further evidence for the capacity of mirror self-recognition in cleaner fish and the significance of ecologically relevant marks
Source: PLoS Biol. 2022 Feb 17;20(2):e3001529. doi: 10.1371/journal.pbio.3001529 (PMC8853551; doi:10.1371/journal.pbio.3001529)
Supplement: Attachment — Submitted filename: Pont by point response letter.docx [file pbio.3001529.s003.docx]

Point by point response letter

From editors

"It must be possible for the authors to give relative frequencies of behavior, i.e. to compare the critical behavior of throat-scraping with the frequency of a few other behaviors in their data and show that *relative* to these other behaviors throat scraping goes up, down, or stays the same. I don't think it will be hard to do, and all we need in the ms is one or two sentences explaining that whatever the claims are for throat-scraping they also hold if we don't measure the behavior by time unit but measure it relative to the rate of other behavior."

From Rev #2

However, you did not address the most crucial point in my last review: Ruling out the likely possibility that increased scores in certain behaviours could have been a side-effect of a marked increase in general behavioural activity. (Aside from some logical reasoning, my scepticism and criticism here is based on experiments I did with fish and mirrors many years ago. Typically, the fish became much more active after getting close to the mirror. As a consequence, a number of behaviours increased in occurrence, including behaviours the fish directed towards themselves. But the proportion of such self-directed behaviours with regard to total behavioural activity did not increase.)

Just to make the point unequivocally clear, a simple example with numbers: Assume the frequency of a certain behaviour, i.e. shaking the head, is normally quite low and the behaviour occurs, on average, every five hours. During an observation period of two hours the measure of occurrence will be zero in most of the cases. Assume now that some factor leads to a fivefold increase in overall behavioural activity. The likelihood for head-shaking will now be about one per hour. Why is it so difficult for you to add general activity data? That could clarify things compellingly.

Without such data, also your point that there was a difference between a brown mark and marks of other colour is not valid. In this case it would be important as well to contextualise the focus data with data on overall activity.

Reply: This time, we clearly understand the reviewer #2’s comment in the first time. So, we reobserved the video recordings of Experiment 1. To show that general activities are not different among the four trails, we measured swimming distance of a fish/ 5 sec 5 times in each trail (n=8 fish). As showing in Line 485-493, we found slight difference in fish swimming speed among the four trials, strongly suggesting that there will not be likely possibility that increased scores of throat-scraping in trial of color-mark with presence of mirror could have been a side-effect of an increase in general behavioral activity.

Line 485-493:

Fish general activities were likely to be not different among four trials in Exp. 1. For example, fish swimming speeds (/sec) were similar among four trails (No treatment: 94.4 mm ± 8.3 SEM; Sham: 97.8 ± 8.9; Color-mark without mirror, 96.5 ± 8.7; Color-mark with mirror, 100.1 ± 9.5), where swimming distance in 5 sec was measured five times / fish in each trial (n = 8 fish). Nevertheless, the swimming speed in marked fish with mirror was slightly but significantly faster than the fish in no treatment (LMM, χ_3_^2^ = 11.22, P = 0.011), suggesting that fish that find a parasite on throat seem to be in a hurry to remove the harm. Anyway, these observations make certain of the results of the mark test.
