# Supplementary material for: Further evidence for the capacity of mirror self-recognition in cleaner fish and the significance of ecologically relevant marks
Source: PLoS Biol. 2022 Feb 17;20(2):e3001529. doi: 10.1371/journal.pbio.3001529 (PMC8853551; doi:10.1371/journal.pbio.3001529)
Supplement: Attachment — Submitted filename: Response to Reviewers.docx [file pbio.3001529.s004.docx]

Response to Reviewers

To Reviewer #2:

It is much appreciated that the authors now have tried to include measures of overall activity in the analysis. However, I am not convinced by these data. What has been added is very little. A good comparison would be based on behavioural units (frequency of biting, fin erection, or the like). That there are differences in swimming activity between conditions in the data now provided is rather opening the possibility that increased activity did play a role in the behavioural activities in front of the mirror than ruling it out.

Res: In the last MS, we added only one behavioral item of swimming speed. Now we show data of not only swimming speed but also frequencies of scraping of body and face, fin spreading and mirror-contingency checking as well as throat scraping and time (sec) in posture reflecting throat in Table 1.

Aside from this, the additional data should be presented in a different way. From my point of view these data represent an essential part of the results, not a minor detail of the methods. Thus, data on overall activity should be reported in the results section. Moreover, based on what I consider good scientific standards, the reporting of results should be separated from interpretation. Biased interpretations should be avoided throughout.

Res: Yes, we agree with Ref 2’s comments. Now these data are not small and should be shown in results and discussion section not in materials and methods. The interpretation and explanations of these data in Table 1 appear in L 151-163 in Results and Discussion section in text. I think it will be of scientific standards. As discussing in text, these additional data support our original interpretation of the results of the mark-test: throat scraping is not byproducts of overall changes in activity patterns but is induced by fish’s motivation to remove it.

Sentences like " ... the swimming speed in marked fish with mirror was slightly but significantly faster than the fish in no treatment (= 11.22, P = 0.011), suggesting that fish that find a parasite on throat seem to be in a hurry to remove the harm ..." are an example of a strongly biased look at the findings. I agree with the academic editor with regard to changing the following sentence. However, I think, the data part has to be moved to the result section and any discussion of it to the discussion part of the "results and discussion section". Furthermore, in my opinion this aspect needs a critical discussion that also takes into account what an alternative explanation would mean to the overall conclusions from the study.

Res: We are sure that the data in Table 1 will reject alternative explanation of throat-mark scraping, that is, throat scraping is a byproduct of a general change in activity patterns. We think that Rev #2’s comments will make our results of the mark-test stronger, and we thanks to him (her).
